# Real-Time Contrast Enhancement to Improve Speech Recognition

**DOI:** 10.1371/journal.pone.0024630

**Published:** 2011-09-19

**Authors:** Joshua M. Alexander, Rick L. Jenison, Keith R. Kluender

**Affiliations:** 1 Department of Speech Language and Hearing Sciences, Purdue University, West Lafayette, Indiana, United States of America; 2 Department of Psychology, University of Wisconsin-Madison, Madison, Wisconsin, United States of America; Hotchkiss Brain Institute, University of Calgary, Canada

## Abstract

An algorithm that operates in real-time to enhance the salient features of speech is described and its efficacy is evaluated. The Contrast Enhancement (CE) algorithm implements dynamic compressive gain and lateral inhibitory sidebands across channels in a modified winner-take-all circuit, which together produce a form of suppression that sharpens the dynamic spectrum. Normal-hearing listeners identified spectrally smeared consonants (VCVs) and vowels (hVds) in quiet and in noise. Consonant and vowel identification, especially in noise, were improved by the processing. The amount of improvement did not depend on the degree of spectral smearing or talker characteristics. For consonants, when results were analyzed according to phonetic feature, the most consistent improvement was for place of articulation. This is encouraging for hearing aid applications because confusions between consonants differing in place are a persistent problem for listeners with sensorineural hearing loss.

## Introduction

This report describes outcomes from normal-hearing (NH) listeners of a real-time signal-processing algorithm, the Contrast Enhancement (CE) algorithm, which was designed generally for communication devices and specifically for hearing aids. The Contrast Enhancement algorithm is so named because it was born out of research that demonstrates how perception of speech is contrastive to the spectral features of neighboring sounds [1]. Classic examples of these phenomena, known generally as contrast effects, take advantage of severe context dependence created by the spatial and temporal overlap of successive articulatory activities that characterize coarticulated speech. It is well known that the second formant frequency (*F_2_*) of vowels is highly influenced by its context when produced between two consonants [2]. For example, because *F_2_* frequency is lower for [b] compared to [d] and [g], vowels produced in a [bVb] context have consistently lower *F_2_* frequency when compared to the same vowels produced in a [dVd] or [gVg] context. Similar observations are made for consonants that are articulated between two vowels [3].

Multiple studies provide evidence that simple processes that perceptually enhance contrastive changes in spectral composition over time can help serve to disambiguate coarticulated speech (for review, see [i]). For example [4], reported that when NH listeners identified synthesized vowels that varied along a series from /U/ to /I/, they were more likely to respond /I/ (higher *F_2_*) when preceded and followed by transitions with a lower *F_2_* onset/offset that acoustically resembled the glide [w]. Conversely, listeners were more likely to respond /U/ (lower *F_2_*) when preceded and followed by transitions with a higher *F_2_* onset/offset that acoustically resembled the glide [j]. These authors wrote: “It is worth reiterating… that mechanisms of perceptual analysis whose operations contribute to *enhancing contrast* in the above-mentioned sense [*i.e.*, the perception of /U/ to /I/] are precisely the type of mechanisms that seem well suited to their purpose given the fact that the slurred and sluggish manner in which human speech sound stimuli are often generated tends to reduce rather than sharpen contrast (p. 842, italics added).” In other words, undershoot in production is compensated for by overshoot in perception, which effectively prolongs the transition slope. It is precisely this sort of spectro-temporal exaggeration of the acoustic spectrum that the CE algorithm attempts to mimic.

Contrast effects like the preceding example are ubiquitous in speech, as they have been reported for a wide variety of phonemes, a wide variety of speech and nonspeech contexts, and a variety of subjects including nonnative listeners, prelinguistic infants, and birds ([5], [6], [7], [8], [9], [10]).

While the specific neural mechanisms responsible for contrast effects are unknown, converging evidence from multiple sources (e.g, [11], [12], [13], [14]) suggests how suppression and adaptation, or higher level processes with similar properties, can support enhanced perception of spectral contrast within and between successive speech segments. If this understanding can be exploited by devices that improve communication, the hypothesized mechanisms behind perceptual contrast need not provide a complete account in order to be very useful. Because coarticulation assimilates the spectrum across time, no matter what the phonetic distinction, enhancement of the spectral differences between successive speech segments (*i.e.*, the contrast) will serve to partially undo such assimilation by perceptually moving sounds away from their neighbors (in this preceding example, along the *F*_2_ dimension). The approach implemented here is to exploit these simple contrastive processes across time through signal processing in a fashion that expands the perceptual space, thereby making adjacent speech sounds more perceptually distinctive.

It has been suggested that their demonstration of vowel aftereffects could be rooted in peripheral sensory adaptation ([11], [12]). One suggestion is that neurons adapt and that the peripheral representation of the added harmonic is made more prominent because neurons tuned to its frequency were not adapted prior to its onset. A number of neurophysiological studies by Delgutte and colleagues support the importance of adaptation in speech perception, especially for enhancing the internal representation of spectral contrast between successive speech segments. [13] for example, notes that peaks in auditory nerve discharge rate correspond to spectro-temporal regions that are rich in phonetic information, that adaptation increases the resolution with which onsets are represented, and that “adaptation enhances spectral contrast between successive speech segments” (p. 512). Likewise, some investigators, e.g. [14], have suggested that rapid adaptation serves mostly to enhance onsets selectively, with suppression being a process through which differences in level of successive spectral regions in complex spectra (e.g., formants in speech signals) are preserved and/or enhanced.

It is well established that listeners with sensorineural hearing loss (SNHL) often do not process frequency-specific information accurately because spectral detail is smeared by broadened auditory filters (e.g., [15], [16], [17], [18], [19], [20], [21]). Loss of sharp tuning in auditory filters generally increases with degree of sensitivity loss and is due, in part, to a loss or absence of peripheral mechanisms responsible for suppression ([22], [23], [24], [25], [26]). Consequently, the peaks of speech for hearing-impaired (HI) listeners are less perceptually distinct and harder to resolve as their internal representations are spread out over wider frequency regions (smeared). This results in less precise frequency analysis, greater confusions between sounds with similar spectral shapes, and subsequently poorer speech recognition ([17], [27], [28], [29], [30]). In this respect, broadened auditory tuning results in an assimilation of the spectrum that defines acoustic features.

Given the evidence that listeners with SNHL experience speech signals with effectively reduced spectral resolution, several attempts (e.g., [31], [32], [33], [34]) have been made to improve speech recognition by sharpening spectral peaks, for example, formant bandwidth narrowing and/or expanding the amplitude of formant peaks relative to surrounding energy. While there are some notable exceptions (e.g., [35]), one significant limitation to most of these techniques is that they depend on block processing which requires relatively long time segments (10–30 ms) and/or significant computational complexity in order to have sufficient frequency resolution for spectral sharpening to occur (see [36], for a technical review). This is unacceptable for real-time applications because research suggests that processing delays >10–15 ms begin to result in disturbances in how hearing aid users perceive their own voice and the speech of others ([37], [38]).

Most attempts at spectral sharpening peaks have met with limited success at best (see [34], for review). For example, some have found improvement for vowels but a decrease in consonant identification (e.g.,[39]) or found significant improvement in vowel identification, but never tested consonant identification ([40]). Several others (e.g., [31], [32], [34]) tested words or sentences, so in cases where no overall improvement was found, it is not clear which parts of speech were improved and which were hindered by the processing. What might be concluded is that spectral sharpening by itself is not effective in alleviating the spectral smearing that accompanies SNHL. Spectral sharpening in isolation might provide limited improvement because enhanced spectral peaks that are close together may still be processed within the same auditory filter, in which case, a means of separating the formants in frequency, enhancing the dynamic spectrum, might be a better option.

The CE algorithm is one attempt to enhance the dynamic spectrum and operates by manipulating both the peak frequency and relative amplitude of moving formants. Perhaps, the most novel feature of the CE algorithm is that it designed to work with multirate filtering techniques to provide real-time signal processing performance, and therefore offers a practical solution to address the consequences of hearing loss. In order to understand how this form of processing affects the perception of specific phoneme classes, experiments in this paper tested the CE algorithm using normal-hearing listeners who identified spectrally smeared consonants (VCVs) and vowels (hVds) in quiet and in noise.

## Methods

### A. Signal Processing

#### 1. Contrast Enhancement Algorithm

**Figure 1:**
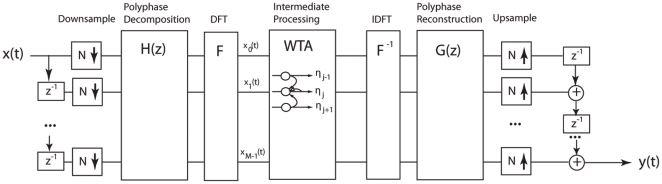
Schematic of the contrast enhancement algorithm. The incoming signal, x(t), is first decomposed into 110.25-Hz wide frequency channels using polyphase decomposition (see text). Intermediate processing incorporates a winner-take-all (WTA) strategy in which channel gain is weighted in a way that simulates a lateral inhibitory network and sharpens spectral contrast. The output signal, y(t), is synthesized by an inverse of the process used to analyze the signal.

Four steps comprise the CE algorithm as shown in Figure 1: (1) signal decomposition into channels, (2) weighting of channel output as a function of time via a dynamic compressive gain function, (3) weighting of channel gain as a function of frequency neighborhoods via a winner-take-all inhibitory network, and (4) signal synthesis. The algorithm described here implemented a sampling rate of 22.05 kHz; however, it can be varied without loss of generality.

A brief description of the filter bank circuit used for contrast enhancement is provided here, a more complete description of the signal processing is provided in Appendix S1. The analysis and synthesis components of the contrast enhancement process use multirate polyphase decomposition and oversampled discrete Fourier transformed (DFT) modulated filters to address problems of aliasing. The input signal, x, is first decomposed into multiple subband channels. Subband channels provide time-varying spectral magnitudes as input to the intermediate processing. The intermediate processing consists of a variant of a winner-take-all (WTA) circuit ([41], [42], [43]), which simulates a biological network of inhibitory sidebands using a form of leaky integration (specifically, a dynamic compressive gain function) to enhance instantaneous spectral contrast and to enhance spectral differences across time within a restricted neighborhood of subbands. Following intermediate processing the stored phase is restored prior to synthesis.

The collective effects of the dynamic compressive gain function and lateral interactions within channel neighborhoods result in a form of energy suppression that progressively sharpens the dynamic spectrum. When an individual channel is relatively high in energy in the past, it will tend to suppress the neighboring channels that are lower in energy. When multiple spectral peaks are within specified neighborhoods of one another, the spectral modes not only sharpen, but also expand with respect to one another through the process of dynamic competition.

**Figure 2:**
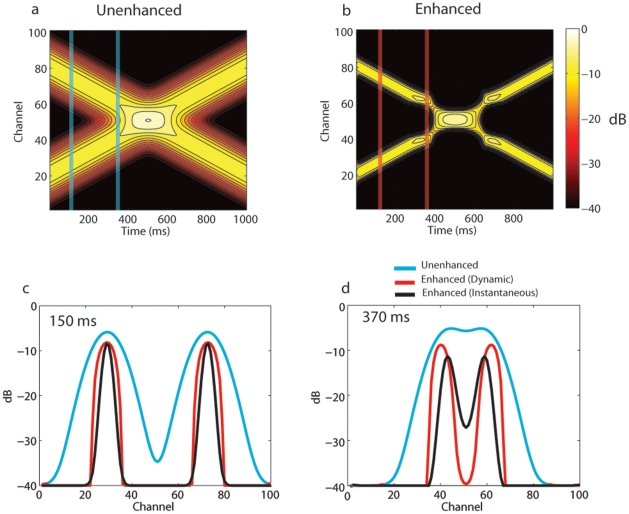
Simulated input to the WTA network. (a) Unenhanced spatiotemporal signal. (b) WTA enhanced spatiotemporal signal demonstrating spectral sharpening and expansion of the position of the spectral peaks as a consequence of dynamic competition between subband channels. (c) Cross-section of channels at 150 ms for unenhanced signals (blue), dynamically enhanced (red), and instantaneously enhanced (black) spatiotemporal signals illustrating spatial (spectral) sharpening. (d) Cross-section of channels at 370 ms for unenhanced (blue), dynamically enhanced (red), and instantaneously enhanced (black) spatiotemporal signals illustrating sharpening and expansion of spectral peaks.

To illustrate the WTA dynamics, an additive pair of signals with spectral Gaussian magnitude distributions across channels was advanced through time across channels 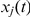 as shown in Figure 2a. The spectrally swept Gaussian functions with each mode (peak) positioned at a location 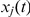 had a constant dispersion (s.d.) of 6 channels with respect to the range of 101 channels. The rate of change for both modes was 60 channel units per second. The modes advance toward one another and crossed at 500 ms. Each image is shown in dB units with respect to the signal maximum. The effects of contrast enhancement are clearly apparent in Figure 2b, as well as the consequential expansion of relative peak energy away from one another as the two modes approach near the point of crossing.

To further visualize these effects, cross-sections are shown in Figures 2c and 2d at 150 ms and 370 ms, respectively. When the modes are within overlapping neighborhoods (Fig. 2c), the mutual inhibition significantly decreased the energy between the competing spectral peaks (Fig. 2c). As the modal components approached one another, the width of each peak in the enhanced signal was further reduced at 370 ms compared to the width at 150 ms. The width of the unenhanced signal 10 dB down from the peak at 150 ms was 18.5 channel units compared to the width of the enhanced signal of 9.7 units. At 370 ms, the two unenhanced modes were at the point of merging. However, the enhanced modes were 8.3 units in width 10 dB down from peak. Not only did the peak widths decrease, but also their frequencies were moved apart. The peak in the unenhanced signal at channel #44 was moved down to channel #41 and the peak at channel #58 was moved up to channel #61.

The spectra represented by black lines in Figs. 2c and 2d demonstrate how the dynamic component of the WTA compares to simple instantaneous enhancement without any history (*i.e.*, without leaky integration). At 150 ms, when the two modes are outside of each other's neighborhood, the dynamic WTA behaves the same as instantaneous enhancement. However, at 370 ms when the two modes begin to cross each other's neighborhood, the advantages of the dynamic circuit become apparent. As expected, instantaneous enhancement is unable to shift the peak frequencies. Furthermore, the spectral contrast (peak-valley difference) is only half of that for the dynamic circuit (about 15 dB compared to 30 dB).

**Figure 3:**
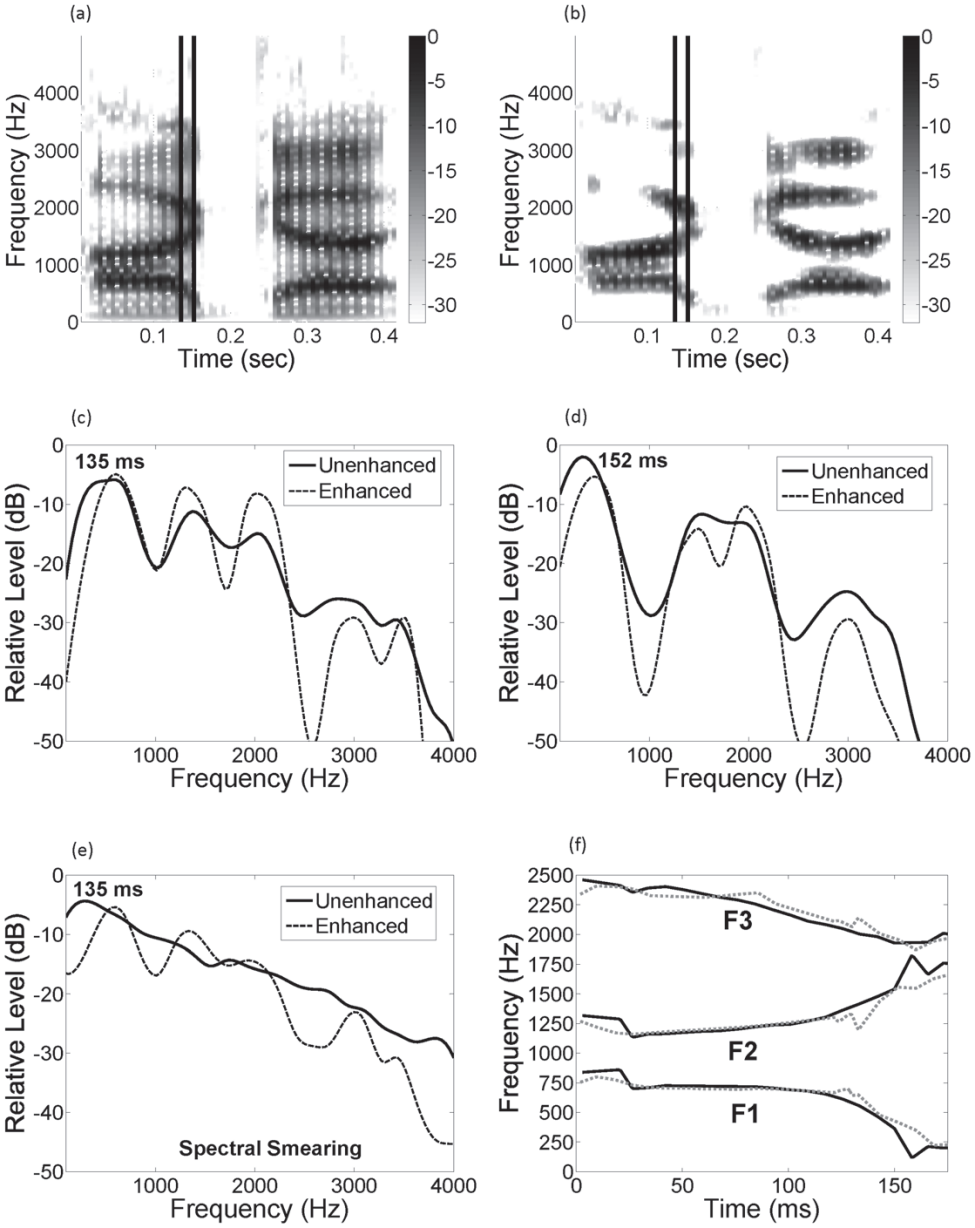
Example of how contrast enhancement and spectral smearing affect the speech spectrum. (a) and (b) Spectrograms of the unenhanced and enhanced VCV /aga/ as spoken by an adult male talker. Vertical lines in the spectrograms correspond to time windows used in (c) and (d), which show spectra for the unenhanced (thick-solid line) and enhanced (thin-dotted line) signals from 16-ms time segments centered at 135 and 152 ms, respectively. (e) Spectral smearing of the unenhanced and enhanced time segments in (c) with a moderate degree of smearing (thick-solid and thin-dotted lines, respectively). (f) Formant peak locations of the stimuli shown in (a) and (b) as derived from linear predictive coding.

Figure 3 demonstrates the effect of the CE algorithm on some of the speech stimuli used in this study. Spectrograms of unenhanced and enhanced speech tokens (/aga/) as spoken by an adult male talker are shown in Figures 3a and 3b, respectively. As with Figure 2b, the increase in relative peak energy at the formant frequencies and the inhibition of energy in between formants are evident in Figure 3b. This is further illustrated in Figures 3c and 3d, which show spectra from 16-ms time segments centered at 135 and 152 ms (2 glottal pulses), during the formant transition from the vowel to the stop closure. Figure 3c again shows how the mutual inhibition of energy between the formants significantly decreases the energy between the peaks. Less obvious is the dynamic shift in formant peak location, with *F*_1_ slightly increasing in frequency and *F*_2_ slightly decreasing in frequency (see following paragraph). Figure 3d shows similar spectral sharpening and formant peak shifting associated with the dynamic inhibitory weighting function [*i.e.*, the right-hand side of Eq. (S1.1) in Appendix S1], but more importantly, also shows how the CE algorithm is capable of separating formants that have merged (in this example, *F*_2_ and *F*_3_). Figure 3e shows the consequence of spectral smearing using a moderate degree of smearing (see next section) on the spectra shown in Figure 3c. Smearing substantially reduces spectral contrast in the signal because relative peak amplitude decreases as the surrounding frequency regions fill with energy. On the other hand, even after enhanced signals are severely smeared, peaks corresponding to formants are modestly preserved.

Figure 3f illustrates the dynamic behavior of the CE algorithm on the speech signal. Formant peak locations of the speech in Figures 3a and 3b, as derived from linear predictive coding are shown. When formant frequencies are relatively constant (<100 ms), the influence of the inhibitory weights tends to be symmetric and spectral sharpening is about equal on the low and high frequency sides of the spectral peaks. However, when the formants change frequency during the transitions, the influence of lateral inhibitory weights is strongest in channels where there was preceding energy, so that spectral sharpening is greatest on the side towards which the formant is moving. Therefore, a formant that transitions from a higher to a lower frequency (in this example, *F*_1_ and *F*_3_) is skewed toward a slightly higher frequency and a formant that transitions from a lower to a higher frequency (in this example, *F*_2_) is skewed toward a slightly lower frequency. The net result is that a pair of diverging formants (in this example, *F*_1_ and *F*_2_) are closer in frequency, which should not be a problem for maintaining instantaneous spectral contrast since they are already moving apart. Conversely, a pair of converging formants (in this example, *F*_2_ and *F*_3_) will be ‘pushed apart’ in frequency, thereby promoting spectral contrast in the dynamic signal.

#### 2. Spectral Smearing

To simulate reduced frequency selectivity associated with SNHL, a technique similar to that described by [44] was used to spectrally smear contrast-enhanced speech, which was then identified by NH listeners. Spectral smearing followed enhancement because alterations of the acoustic signal are ultimately disrupted by cochlear processing. Two degrees of smearing were used: moderate and severe. See Appendix S2 for full details. Spectral smearing followed contrast enhancement.

### B. Listeners and Ethics Statement

Across all conditions, 166 normal-hearing listeners were recruited. Listeners for all experiments were undergraduate students from the University of Wisconsin- Madison who participated for course credit. Written consent obtained for all listeners. Procedures and use of human subjects for this study were approved by the Institutional Review Board of the University of Wisconsin-Madison (protocol number SE-2004-0612). No listener participated in more than one condition for each stimulus type (consonants and vowels). All reported that they were native speakers of American English and had normal hearing. Listeners completed the experiments while seated in a double-walled sound chamber.

### C. Speech Material

Consonant recognition was tested using 60 vowel-consonant-vowel (VCV) syllables formed by combining 20 consonants (/p, t, k, b, d, g, f, θ, s, ∫, v, z, 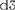, t∫, m, n, l, r, w, y/) and three vowels (/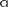, i, u/). The 60 VCVs were recorded with 16-bit resolution and 22.05 kHz sampling rate from three adult male and three adult female talkers, all with Upper Midwestern accents. To simulate the bandwidth of a typical hearing aid, each VCV was low-pass filtered at 4.8 kHz with a 100-order FIR filter.

Vowel recognition was tested using the twelve /h/-vowel-/d/ (hVd) syllables from the [45] database (/i, 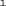, e, ε, æ, 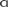, 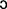 , o, 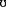, u, 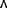, 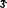). Fourteen talkers (4 men, 4 women, 3 boys, and 3 girls) from the 139-talker database (Upper Midwestern accents) were selected. Based on data from 20 NH adults [45], the chosen talkers all had overall identification rates of at least 97.5% correct and individual token identification rates of at least 90% correct. The tokens were upsampled from 16.0 to 22.05 kHz and low-pass filtered in the same manner as the consonants stimuli.

### D. Procedure

Stimuli were presented monaurally with 24-bit resolution and 22.05 kHz sampling rate through BeyerDynamic DT150 headphones. Consonant stimuli were spoken by two adult male and two adult female talkers (80 total for each vowel context). Conditions were blocked by vowel context, the order of which was determined by a random number generator for each listener. Vowel stimuli were spoken by 10 talkers (120 total): 3 men, 3 women, 2 boys, and 2 girls. The order of stimulus presentation for each condition and for each listener was also random. Following the stimulus presentation, listeners identified what they thought they heard by using a computer mouse to click the place on a grid display that corresponded to their response.

### E. Conditions

Baseline performance for speech materials without spectral smearing or enhancement was measured for 45 NH listeners (3 groups of 15) using the methods outlined above. One group of listeners identified VCVs and hVds in quiet and another group identified them in pink noise at 6 dB signal-to-noise ratio (SNR) for the VCVs and at 0 dB SNR for the hVds. Approximately half of the listeners identified VCVs first and half identified hVds first. A higher SNR was initially used for VCVs because pilot testing with spectral smearing indicated high error rates when SNR was further decreased. A third group of listeners later identified hVds at 6 dB SNR so that performance for the different speech materials could be compared at the same SNR.

Ten groups of NH listeners identified spectrally smeared speech materials. With one exception, each group consisted of 12 individuals. Half of the listeners in each group identified unenhanced stimuli first followed by enhanced stimuli and the other half identified enhanced stimuli first followed by unenhanced stimuli. Four groups identified VCVs: two in quiet and two in pink noise at 6 dB SNR, each with moderate and severe amounts of smearing. Unintentionally, a thirteenth listener was recruited in the group tested on VCVs in quiet with severe smearing. Six groups identified hVds: two in quiet, two in pink noise at 6 dB SNR, and two in pink noise at 0 dB SNR. As with VCVs, half of the two groups identified stimuli with moderate smearing and the other half with severe smearing.

## Results

### A. Consonant Stimuli

**Figure 4:**
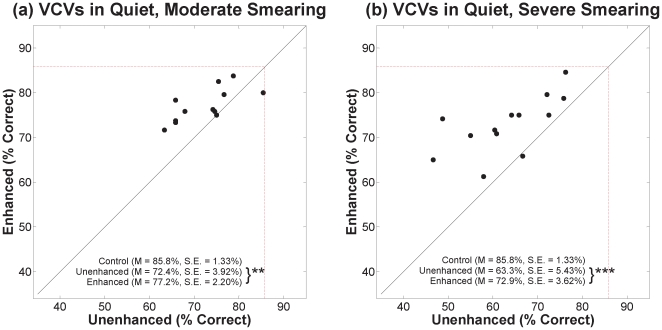
Scatter plots of percent correct for VCVs in quiet. Results for moderate and severe degrees of smearing are in panels (a) and (b), respectively. Percent correct for unenhanced speech is represented along the abscissa and percent correct for contrast-enhanced speech is represented along the ordinate. The red dashed box represents the percent correct for the control speech (no smearing, no enhancement). The mean and standard errors for each condition are displayed on the graph. Asterisks indicate the significance level for paired t-tests [** for *p*≤0.01 and *** for *p*≤0.001].

**Figure 5:**
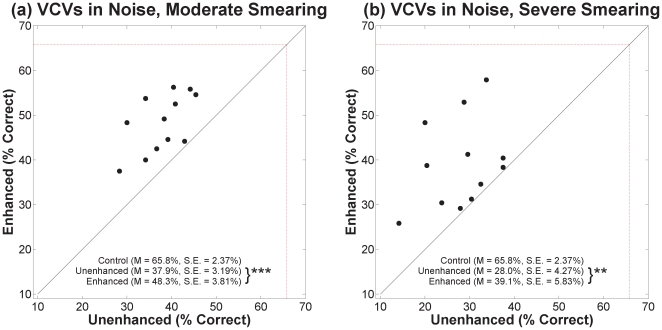
Scatter plots of percent correct for VCVs in noise. See Figure 4 legend.

Scatter plots of percent correct for VCVs in quiet and in noise are shown in Figures 4 and 5, respectively. Panels (a) and (b) of each figure show results for moderate and severe degrees of smearing, respectively. Results for unenhanced speech are represented along the abscissa and results for contrast-enhanced speech are represented along the ordinate. The red dashed box represents the percent correct for the control speech (no smearing, no enhancement). The mean and standard errors for each condition are displayed on the graph. Asterisks next to the descriptive statistics indicate level of significance corresponding to paired t-tests with 11 degrees of freedom (12 for severe smearing in quiet), [* for *p*≤0.05, ** for *p*≤0.01, and *** for *p*≤0.001].

Here, and throughout, identification rates were transformed to rationalized arcsine units [46] before statistical analyses were conducted (plots show un-transformed values). Contrast-enhanced speech was correctly identified at a significantly higher rate than unenhanced speech for each degree of smearing, in quiet and in noise. For VCVs in quiet, a mixed-design analysis of variance (ANOVA) with enhancement as the within-subjects variable and degree of smearing as the between-subjects variable (Table 1) revealed significant main effects for contrast enhancement and for degree of smearing, but no significant interaction. Outcomes for VCVs in noise yielded the same pattern. Lack of an interaction in each case indicates that benefit from enhancement did not depend significantly on the degree of spectral smearing.

**Table 1:** Anova results.

Condition	Enhancement	Smear	Interaction
VCVs in Quiet	*F*(1,23) = 35.4^***^	*F*(1,23) = 6.9^*^	*F*(1,23) = 3.6, *^N.S.^*
VCVs (6 dB SNR)	*F*(1,22) = 40.6^***^	*F*(1,22) = 14.3^***^	*F*(1,22)<1.0, *^N.S.^*
hVds in Quiet	*F*(1,22) = 19.6^***^	*F*(1,22) = 9.7^**^	*F*(1,22) = 2.7, *^N.S.^*
hVds (6 dB SNR)	*F*(1,22) = 60.9^***^	*F*(1,22) = 7.5^**^	*F*(1,22)<1.1, *^N.S.^*
hVds (0 dB SNR)	*F*(1,22) = 37.7^***^	*F*(1,22) = 8.6^**^	*F*(1,22)<1.0, *^N.S.^*

For VCVs and hVds in quiet and in noise, outcomes for mixed-design ANOVAs with enhancement as the within-subjects variable and degree of smearing as the between-subjects variable. Asterisks indicate level of significance [* for *p*≤0.05, ** for *p*≤0.01, and *** for *p*≤0.001] and *N.S.* indicates a non-significant result.

To see if benefit of contrast enhancement depended on the addition of noise, a between-subjects ANOVA was conducted with the difference in performance between enhanced and unenhanced conditions as the dependent variable and quiet vs. noise as the independent variable (collapsed across both degrees of smearing). There was no significant difference in benefit between the quiet conditions (*M* = 7.3%, *SE* = 1.33%) and the noise conditions (*M* = 10.3%, *SE* = 1.69%) [*F*(1,47) = 2.3, *p*>0.05].

To understand how the CE algorithm influenced the perception of different phonetic features, feature errors of the consonant stimuli were analyzed using sequential information transfer analysis or SINFA [47], [48]. Confusion matrices and a list of distinctive features (voicing, nasality, manner, and place of articulation) associated with each phoneme serve as input to SINFA. SINFA output includes proportion of information transferred, IT, for each feature (information received divided by information transmitted). Tables 2 and 3 show IT for each distinctive feature for the quiet and noise conditions, respectively. Spectral smearing was effective at degrading most features of the speech signal. For all four experimental conditions, contrast-enhanced speech improved IT for place of articulation in quiet and in noise and improved IT for manner in the noise conditions only.

**Table 2:** Feature analyses for speech in quiet.

Condition	Voicing	Nasality	Manner	Place	Total IT
Control	0.80	0.93	0.82	0.74	3.56
Unenhanced Moderate	0.77	0.77	0.68	0.49	2.94
Enhanced Moderate	0.85	0.72	0.68	0.58	3.10
Unenhanced Severe	0.76	0.67	0.58	0.35	2.57
Enhanced Severe	0.79	0.71	0.62	0.53	2.88

Proportion of information transferred, IT, for each phonetic feature (information received divided by information transmitted) for the VCVs presented in quiet.

**Table 3:** Feature analyses for speech in noise.

Condition	Voicing	Nasality	Manner	Place	Total IT
Control	0.66	0.52	0.54	0.47	2.55
Unenhanced Moderate	0.48	0.41	0.24	0.15	1.49
Enhanced Moderate	0.47	0.38	0.32	0.30	1.76
Unenhanced Severe	0.34	0.24	0.15	0.10	1.06
Enhanced Severe	0.36	0.28	0.21	0.21	1.35

Proportion of information transferred, IT, for each phonetic feature (information received divided by information transmitted) for the VCVs presented in noise.

To evaluate if the benefit of contrast enhancement depended on talker gender, differences in error rates between unenhanced and enhanced speech for each gender were submitted to a repeated-measures ANOVA. For each feature and each condition, there was no significant difference in benefit of enhancement between men and women talkers (*p*>0.05).

### B. Vowel Stimuli

**Figure 6:**
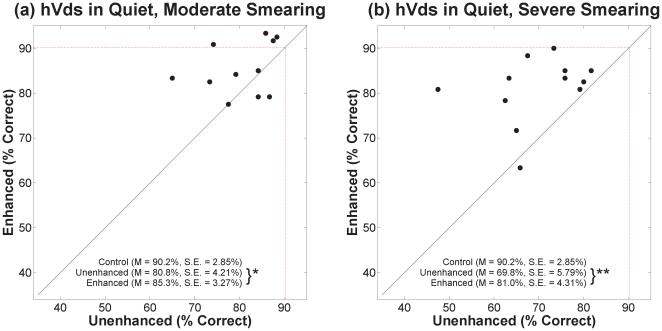
Scatter plots of percent correct for hVds in quiet. See Figure 4 legend. Asterisks indicate the significance level for paired t-tests [* for *p*≤0.05 and ** for *p*≤0.01].

**Figure 7:**
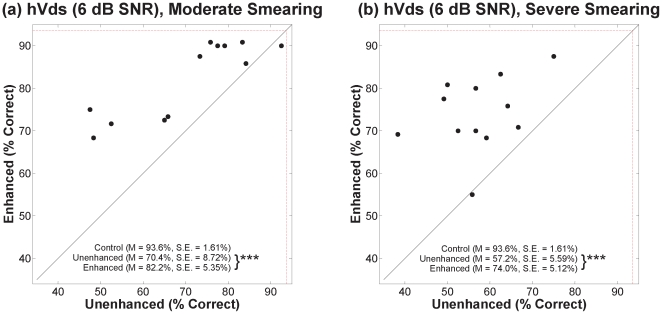
Scatter plots of percent correct for hVds in 6 dB SNR noise. See Figure 4 legend.

**Figure 8:**
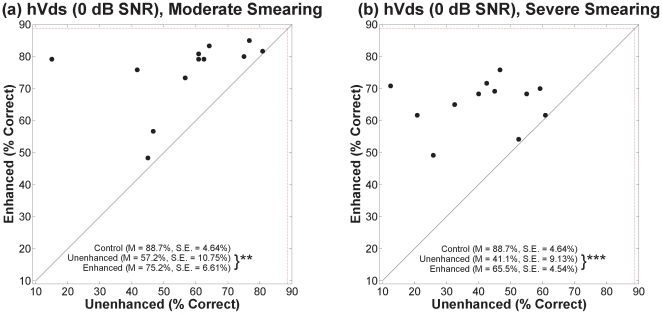
Scatter plots of percent correct for hVds in 0 dB SNR noise.

Figures 6, 7, and 8 show percent correct for hVds in quiet and in the two noise conditions in the same format as the VCVs. Contrast-enhanced speech was correctly identified at a significantly higher rate than unenhanced speech for each degree of smearing, in quiet and in both noise conditions. For hVds in quiet, a mixed-design ANOVA (Table 1) revealed significant main effects for contrast enhancement and for degree of smearing, but no significant interaction. Outcomes for hVds in both noise conditions yielded the same pattern. As with VCVs, lack of a significant interaction in each hVd condition indicates that benefit from enhancement did not depend on the degree of spectral smearing.

To learn whether the benefit of contrast enhancement depended on the addition of noise, a between-subjects ANOVA was conducted with the difference in performance between enhanced and unenhanced conditions as the dependent variable and the amount of noise (quiet, 6 dB SNR, and 0 dB SNR) as the independent variable (collapsed across both degrees of smearing). There was a significant difference in benefit between the conditions [*F*(2,69) = 5.5, *p*<0.01]. Tukey post-hoc tests revealed that benefit for the 0 dB SNR condition, (*M* = 21.2%, *SE* = 3.48%) was significantly greater (*p*<0.05) than benefit for the quiet condition (*M* = 7.9%, *SE* = 1.99%). Benefit for the 6 dB SNR condition (*M* = 14.3%, *SE* = 1.98%) was not significantly different from that for the other two conditions.

To determine whether the benefit of contrast enhancement depended on talker group, differences in error rates between unenhanced and enhanced speech for each group were analyzed using a repeated-measures ANOVA with talker group as the within-subjects variable. There was no significant effect of talker group for any condition (*p*>0.05). Lack of a significant effect in every case indicates that benefit from enhancement for vowel stimuli did not depend substantially on talker characteristics, namely, fundamental frequency and formant spacing.

## Discussion

Large impairments in consonant identification associated with spectral smearing were improved when speech in quiet and in noise was first processed with the CE algorithm. The most consistent improvement across all four conditions (two degrees of smearing in quiet and in noise) was for place of articulation. This finding is encouraging for hearing aid applications because, as [49] point out, “The frequency of place errors among hearing-impaired listeners is a consistent finding throughout the literature, despite differences in materials, talkers, and experimental procedures” (p. 147). The large increase in place errors associated with spectral smearing and the resultant improvement associated with contrast enhancement is consistent with the importance of spectral information (e.g., second formant transitions) for cueing differences in place of articulation. A significant decrease in manner errors with enhancement occurred only for VCVs in noise. Noise contributes to the reduction of spectrally specific information and disrupts other cues used to distinguish manner (e.g., frication, temporal envelope). Therefore, information preserved by contrast enhancement, specifically formants and formant transitions, might have been of additional benefit to listeners when noise was present. A comparison of feature errors between unenhanced and enhanced speech across talker gender did not reveal any significant differences. A similar comparison across vowel context did not reveal any consistent pattern. It is important to note that, while there might have been significant differences in absolute error rates across talker gender or vowel context (e.g., higher error rates for female talkers and for the /i/ context), when unenhanced and enhanced spectrally smeared speech were compared to the control or to each other, these differences were not statistically significant.

Contrast enhancement also significantly improved identification of spectrally smeared vowels in quiet and in noise. As with consonants, improvement did not depend on the degree of spectral smearing or talker characteristics. The latter is an important finding because it indicates that success of the CE algorithm does not depend on harmonic spacing or formant separation in the vowel space, both of which increase with women and child talkers owing to higher fundamental frequencies and shorter vocal tracts, respectively. Improvement in vowel identification did depend on the amount of noise, with significantly greater improvement for hVds in noise at 0 dB SNR than in quiet. This result is expected because noise effectively reduces the amount of spectral contrast in the signal by filling the valleys between the peaks with energy. In addition, noise reduces the salience of other speech cues, which increases the importance of spectral cues made more salient with CE. Because VCVs in noise were only tested at 6 dB SNR, it is unknown whether benefit from the CE algorithm for consonant identification would likewise increase with more challenging noise levels.

The increased benefit from the CE algorithm with decreasing SNR is encouraging because several noise reduction algorithms in hearing aids rely on modulation depth and/or modulation frequency for estimating the presence of speech in a noisy signal, and both indicators decrease in sensitivity and specificity as SNR decreases. This suggests that another application of the CE algorithm could be the front-end of a two-stage noise reduction scheme. That is, the CE algorithm could be used to improve the representation of speech in the acoustic signal, which would feed into a noise reduction algorithm that selectively attenuates frequency bands with detrimentally low SNR.

One limitation of this study is that the reduction of spectral contrast by employing a fixed-window FFT analysis does not fully represent what the auditory system actually does. As mentioned in the Introduction [2], another important factor that determines spectral contrast is temporal dynamics, such that slurred and sluggishly produced formant transitions are effectively prolonged by perceptual mechanisms operating in time. It is unknown what effect SNHL has on these mechanisms beyond simple filter broadening. Furthermore, for the auditory system, the contrast between spectral peaks and valleys is different from what is visually apparent in the spectral analysis. One reason for this is that peaks mask not only valleys but also nearby peaks, and they do so in an asymmetric way. Therefore, differences that are visible when comparing spectrograms of coarticulated utterances may be diminished by mechanisms operating in time.

Some caution is warranted in extrapolating from NH listeners with simulated hearing loss via spectral smearing, because there are additional factors that contribute to SNHL. The decrease in performance for NH listeners is primarily attributed to the smoothing of the spectral envelope introduced by smearing and to the addition of noise introduced by partial randomization of phase. Moore and colleagues [50] identify at least two other consequences of reduced frequency selectivity for listeners with SNHL that are not mimicked by spectral smearing. The first is that sinusoidal signals will generate broader excitation patterns, thereby limiting the ability of HI listeners to resolve the harmonic structure associated with voiced speech. The second is that the timing information at the output of the auditory filters will be distorted. While the most ecologically valid test will involve hearing-impaired individuals listening to contextually meaningful sentences, our methods were chosen in order to control for subject variables (which vary tremendously with a heterogeneous clinical population) and to provide the most analytically useful dataset. One nice feature about the CE algorithm is the ability to customize the time constants, degree of enhancement, and frequency extent of lateral inhibition as a function of frequency. Studies involving customization of the CE algorithm for individual hearing-impaired listeners are underway.

Despite these limitations, the overall results of this study indicate that when the speech spectrum is uniformly smeared across frequency, the CE algorithm, which enhances spectral differences within and across successive spectral segments, is successful in partially restoring intelligibility for NH listeners. These results are promising and, as discussed earlier, suggest that the CE algorithm could be most beneficial when used to augment modern digital hearing aid applications.

## Supporting Information

Appendix S1 **Contrast Enhancement Algorithm.** Description of the signal processing for real-time contrast enhancement.
(PDF)

Appendix S2 **Spectral Smearing.** Description of the technique used to spectrally smear contrast-enhanced speech.
(PDF)
