# Supplementary material for: Real-Time Contrast Enhancement to Improve Speech Recognition
Source: PLoS One. 2011 Sep 19;6(9):e24630. doi: 10.1371/journal.pone.0024630 (PMC3176230; doi:10.1371/journal.pone.0024630)
Supplement: Appendix S1 — Contrast Enhancement Algorithm. Description of the signal processing for real-time contrast enhancement. (PDF) [file pone.0024630.s001.pdf]

APPENDIX S1: CONTRAST ENHANCEMENT ALGORITHM

1. Multirate Processing

Multirate processing effectively distributes a window of sample points in time over the channels of the filter bank resulting in a significant improvement in processing time as a consequence of sampling decimation. Filter banks are widely used in digital signal processing and subband techniques are useful for processing each band independently. Multirate techniques can be used to significantly reduce the computational demands of implementation [S1]. A subband scheme utilizes an analysis filter bank that splits the input into a set of M narrow-band signals that are typically also downsampled (decimated) by some factor N, leading to more efficient processing. Intermediate processing can be performed and the constituents subsequently combined using a synthesis filter bank that is upsampled (interpolated) by a factor of N. If no intermediate processing is performed, it is generally acknowledged that the input can be perfectly reconstructed at the output. In principle, the implementation can be decimated up to the chosen number of subbands, which is commonly referred to as a critically downsampled filter bank (i.e., $M=N$). The problem with critical decimation is that significant aliasing distorts the subband signals. A common solution is to oversample the subbands (i.e., $N > M$), which adds additional computational cost, but resolves the aliasing problem.

Oversampling addresses the common aliasing problem across channels in multirate processing. The method for decomposition is largely independent of the internal processing between analysis and synthesis. It is important that leakage between channels be minimized. However, our choice of polyphase decomposition was largely based on efficiency and other filter-bank-based decomposition approaches offer comparable channel separation. Consequently, only a brief description of the present decomposition approach is presented. Further detail can be found in standard multirate texts [S1], [S2], [S3].

The M subband filters are derived by frequency shifting a well-constructed prototype low-pass filter h[t]. Polyphase decomposition groups the analyzing prototype filter h[t] into M subsequences prior to Fourier transformation [S4], [S5]. This segmented representation allows rearrangement of the filtering computations and increases the speed of processing approximately M-fold.

The circuit used for the CE algorithm (Fig. 1 in the article) contains M = 100 subband channels with 110.25 Hz bandwidth, and a decimation factor N = 25, resulting in fourfold oversampling. The M x N polyphase decomposition matrix H(z) contains the components of h[t] and the M x M DFT matrix F generates a decomposed complex signal for intermediate processing. The synthesis bank contains the inverse DFT matrix and the reconstruction matrix G(z).

2. Subband signal processing

The oversampled polyphase filter bank downsamples by a factor of 100, and then upsamples (oversamples) by a factor of 4 resulting in a sampling rate $FS_{subband} = 882$ Hz. The magnitude of each subband signal $x_j(t)$ feeds into the WTA circuit where the vector element $\eta_j(t)$ corresponds to the state activity of the jth subband channel node in the discrete time period index t (see Fig. 1 in the article). Self-feedback excites with weight w_j^j and neighboring nodes inhibit with weights w_j^k. A nonlinear convex loss function $\gamma[\eta_j(t)] = \tanh^{-1}[\eta_j(t)]$ contributes the necessary nonlinear compression that generates competition between subband channels, as well as ensuring stability of the network. The WTA circuit was implemented as the following difference equation

$$\eta_j(t) = \eta_j(t-1) + \beta\left\{x_j(t-1) + w_j^j\eta_j(t-1) + \sum_{k \neq j} w_j^k\eta_k(t-1) - \gamma\left[\eta_j(t-1)\right]\right\}. \quad \text{(S1.1)}$$

The parameter β controls the history decay for each advancement in time, and therefore, the network memory. Due to the system nonlinearities and contributions of neighboring subbands it is

difficult to derive an analytical impulse response, however an approximation can be made under certain constraints. The first order Taylor series approximation of $\gamma\left[\eta_j(t)\right]$ near zero yields $\gamma\left[\eta_j(t)\right] = \eta_j(t)$, and ignoring the contribution of the neighboring subbands simplifies to the following linear difference equation

$$\eta_j(t) = \left(1 + \beta w_j^j - \beta\right)\eta_j(t-1) + \beta x_j(t-1) \quad \text{(S1.2)}$$

The inverse z-transform of the transfer function of this difference equation yields the impulse response function for the jth subband.

$$\beta\left(1 + \beta w_j^j - \beta\right)^t u(t) \quad \text{(S1.3)}$$

where $u(t)$ is the unit step function. Solving for the time at which the response decays to e^{-1} of the initial value generates

$$\tau = -\log\left(1 + \beta w_j^j - \beta\right)^{-1} / FS_{subband} \quad \text{(S1.4)}$$

Although greatly oversimplified, this function does provide a first order approximation of the duration of the network memory.

The network at steady state (i.e. when $\eta_j(t) = \eta_j(t-1)$) has the following characteristics:

Let

$$I_j(t) = x_j(t) + w_j^j \eta_j(t) + \sum_{k \neq j} w_j^k \eta_k(t) \quad \text{(S1.5)}$$

which implies

$$I_j(t) = \gamma\left[\eta_j(t)\right] . \quad \text{(S1.6)}$$

The solution for the output $\eta_j(t)$ over the domain 0 to 1 is therefore

$$\eta_j(t) = \gamma^{-1}\left[I_j(t)\right] \tag{S1.7}$$

where $\gamma^{-1}\left[I_j(t)\right] = \tanh\left(I_j(t)\right)$ and ensures a range restricted to 0 and 1 so long as $I_j(t)$ is positive (see Fig. S1a). $\gamma^{-1}\left[I_j(t)\right]$ reveals the implicit nonlinear input-output compression (Fig. S1b). However, the function is not instantaneous because of the dynamic implementation, and therefore distortion products are minimized. The first derivative over the restricted domain is

$$\frac{d\gamma\left[\eta_j(t)\right]}{d\eta_j(t)} = \frac{1}{1-\eta_j(t)^2} \tag{S1.8}$$

which implies $\gamma\left[\eta_j(t)\right] - \gamma\left[\eta_j(t-1)\right] > \eta_j(t) - \eta_j(t-1)$. However, $\gamma\left[\eta_j(t)\right]$ in this case approaches infinity as $\eta_j(t)$ approaches 1. Consequently, $\eta_j(t)$ is set to 0 when $\eta_j(t) < 0$.

The neighborhood of channels symmetrically surrounding each subband channel was set to +/- 20 channels (+/- 2205 Hz). Neighborhood weights w_j^k were constructed as off diagonal elements of a Toeplitz matrix [S6] set to a constant negative value (-0.1) within the neighborhood and zero elsewhere (see Fig. S1c). The self-exciting weight w_j^j on the diagonal was set to a positive value (+0.5). The history weighting β was set to 0.25, which under the analytical constraints stated earlier would correspond to an approximate time constant $\tau = 8.5$ms. This calculation is only relevant when the algorithm is operating in the linear region and a given subband is not under the influence of neighboring subbands. Under certain restrictions, it is possible to prove that one subband state activity $\eta_j(t)$ in the neighborhood will remain above an arbitrary threshold and the others below threshold [S7]. In essence, the subband channels activities “compete” on a moment-by-moment basis and subsequently combined together with phase information by the synthesis filter bank to yield the contrast-enhanced signal (Fig. 1 in the article).

REFERENCES

[S1] Vaidyanathan PP (1990) Multirate digital filters, filter banks, polyphase networks, and applications - a tutorial. Proc. IEEE 78: 56-93.

[S2] Vaidyanathan PP (1993) Multirate Systems and Filter Banks. Englewood Cliffs, N.J.: Prentice Hall.

[S3] Fliege N (1994) Multirate Digital Signal Processing: Multirate Systems, Filter Banks, Wavelets. Chichester, NY: Wiley.

[S4] Cvetkovic Z, Vetterli M (1998) Oversampled filter banks. IEEE Trans. Signal Process 46: 1245-1255.

[S5] Eneman K, Moonen M (2001) DFT modulated filter bank design for oversampled subband systems IEEE Trans. Signal Process. 81: 1947-1973.

[S6] Golub GH, Van Loa CF (1996) Matrix computations, 3rd ed. Baltimore: Johns Hopkins University Press.

[S7] Kohonen T (1997) Self-Organizing Maps. Berlin, NY: Springer.

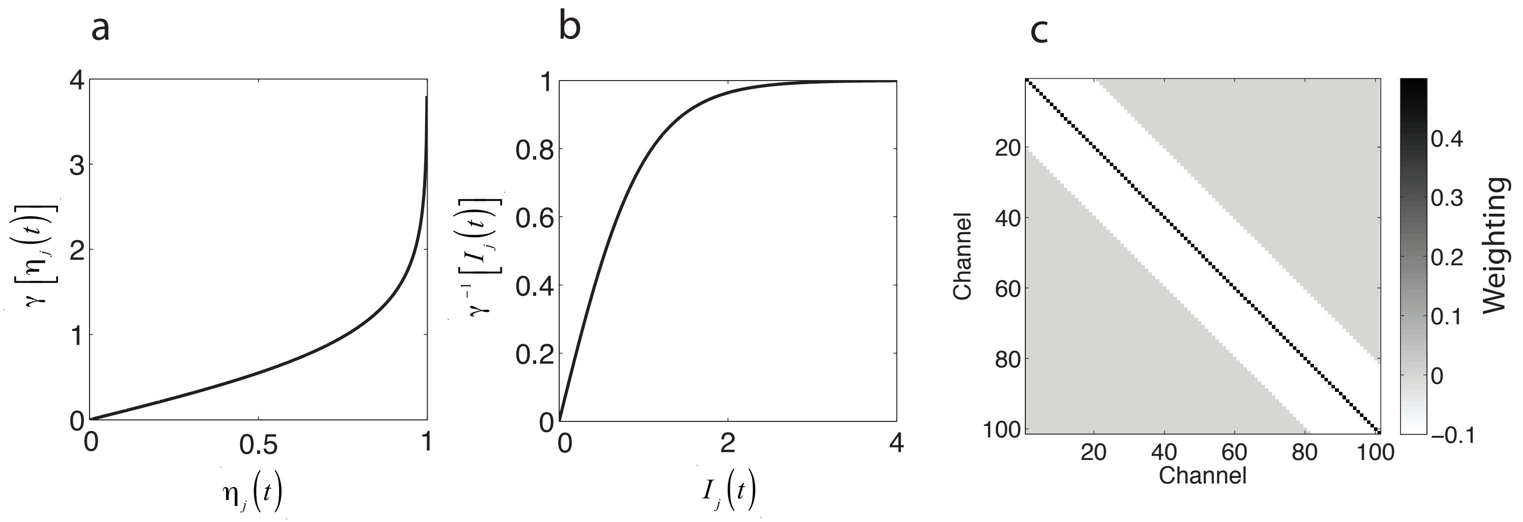

Fig. S1. Contrast enhancement algorithm details. (a) Convex loss function used in the implementation of the WTA circuit. (b) Steady-state input-output compression function resulting from the dynamic application of the convex loss function. (c) Toeplitz matrix implementation of neighborhood weights.
