# Supplementary material for: Real-Time Contrast Enhancement to Improve Speech Recognition
Source: PLoS One. 2011 Sep 19;6(9):e24630. doi: 10.1371/journal.pone.0024630 (PMC3176230; doi:10.1371/journal.pone.0024630)
Supplement: Appendix S2 — Spectral Smearing. Description of the technique used to spectrally smear contrast-enhanced speech. (PDF) [file pone.0024630.s002.pdf]

APPENDIX S2: SPECTRAL SMEARING

Techniques similar to those described by [S8] were used to spectrally smear the speech stimuli. First, the source signal (22.05 kHz sampling rate and 16-bit resolution) was divided into 256-sample (11.6 ms), Hamming-windowed segments which were zero-padded with 128 samples at the beginning and end. Magnitude and phase components of each segment were derived from a 512-point FFT. Next, the magnitude spectrum (a column vector) was multiplied by a smearing matrix (described next) and phases of individual FFT components were jittered by adding a deviate from a uniform random distribution with bounds at +/- 0.2π. The time waveform resulting from inverse FFT of the smeared spectrum was scaled to the same amplitude as the unsmeared source segment. The output time waveforms were then added together using 75% overlap [S9].

For each frequency component of the input spectrum, an auditory filter function was computed and stored as a row in a 256-by-256 matrix. Auditory filter functions were derived using the one-parameter form of the *roex*(*p*) filter [S10] and were divided by the equivalent rectangular bandwidth (ERB) of the filter center frequency [ERB = 24.7(4.37F + 1), where F is the center frequency of the filter (in kHz)] to remove the upward shift in spectral tilt that results when computing excitation patterns (see [S8] or [S11]). The parameter *p* in the *roex* model that determines the sharpness of the filter was set equal to four times the filter center frequency divided by its ERB to approximate the width of a typical auditory filter for a NH listener ([S11]). Widened auditory filter functions were created by dividing this value for *p* by 6 for moderate smearing or by 9 for severe smearing. The smearing matrix was derived by multiplying the inverse of the matrix for a normal auditory filter bank with the matrix for the widened auditory filter bank. This has the effect of reducing the amount of spectral smearing in the acoustic signal by an amount roughly equivalent to the spectral smearing that will occur when the signal is processed by a NH listener's auditory filters ([S8]).

The spectral smearing procedure used here differs from that of [S8] who used an 8-ms sample window (128-point window size with a 16 kHz sample rate) and who smeared the power spectrum and preserved phase. The effect of smearing the magnitude spectrum instead of the power spectrum (magnitude squared) is to reduce the amount of effective smearing. (Smearing the power spectrum is the correct method. Due to a programming error, the magnitude spectrum was smeared.) The procedure of randomizing phase is closer to the one used by of [S12] to introduce noise and suppress re-emerging of peaks caused by overlapping segments. [S12] completely randomized the phases of small components, and left the phases of peak components untouched (like [S8]).

In order to estimate the effect this processing had on the filter bandwidth relative to normal, acoustic analyses were conducted using a short segment of the vowel /ɛ/ from one of the adult male talkers in the study. This segment was processed through all the conditions, as described above. Each processed segment was then divided into eight 512-point frames. The estimated excitation pattern for normal auditory filters for each frame was then computed. Recall that the smearing involved 'correcting' for the normal-hearing auditory filters so that when they were presented to the listeners, the excitation pattern would simulate that for the impaired ear. Each frame of the smeared speech (normal excitation pattern) was compared to the same frame of the original vowel after going through an impaired excitation pattern. An iterative algorithm was used to find the amount by which the tuning for the original vowel had to be broadened to match that of the smeared vowel. Because smearing involved partial phase randomization in an attempt to de-correlate overlapping segments, this procedure was repeated on 10 different phase randomizations (*i.e.*, the same vowel was processed through the smearing algorithm 10 times).

For comparison, Figure S2 shows the excitation pattern of the normal, un-smeared vowel. Figures S3 and S4 show example frames of the acoustic analyses for the vowel in quiet and in noise,

respectively. The blue bars show the excitation patterns of the smeared vowels and the red lines show the least-squares fit of the impaired excitation pattern for the normal vowel. Panels (a) and (c) of each figure show excitation patterns for the unenhanced vowels while panels (b) and (d) show the excitation patterns for the enhanced vowels. Panels (a) and (b) of each figure represent a moderate amount of spectral smearing and panels (c) and (d) represent a severe amount of smearing.

The values in the Table S1 represent the amount by which the excitation pattern for the original vowel had to be broadened (*re*: normal) in order to match the individual smeared frames. The numbers in the parentheses represent the standard error across the 10 repetitions (phase randomizations), each of which were averaged across their eight frames. Moderate smearing for vowels in quiet corresponded to an excitation pattern with an auditory filter bandwidth 3.49 times wider than normal, while severe smearing corresponded to 4.91 times wider than normal. Pink noise at 6 dB SNR only modestly increased these values. Enhancement reduces the broadened excitation pattern by about 50%. Finally, phase randomization significantly widened the excitation patterns of the individual frames compared to smearing without partial phase randomization, but not by a substantial amount (M = 3.42 times normal [$t(9) = 3.0$, $p < 0.05$] and M = 4.78 times normal [$t(9) = 4.5$, $p < 0.01$] for moderate and severe smearing, respectively, of unenhanced versions of the vowels in quiet).

REFERENCES

[S8] T. Baer and B. C. J. Moore (1993) "Effects of spectral smearing on the intelligibility of sentences in noise," *J. Acoust. Soc. Am.* 94, 1229-1241.

[S9] J. B. Allen (1977) "Short term spectral analysis, synthesis, and modification by discrete Fourier transform," *IEEE ASSP* 25, 235-238.

[S10] R. D. Patterson, I. Nimmo-Smith, D. L. Weber, and R. Milroy (1982) “The deterioration of hearing with age: Frequency selectivity, the critical ratio, the audiogram, and speech threshold,” *J. Acoust. Soc. Am.* 72, 1788-1803.

[S11] B. C. J. Moore, B. R. Glasberg, and A. Simpson (1992) “Evaluation of a method of simulating reduced frequency selectivity,” *J. Acoust. Soc. Am.* 91, 3402-3423.

[S12] J. Lyzenga, J. M. Festen, and T. Houtgast (2002) “A speech enhancement scheme incorporating spectral expansion evaluated with simulated loss of frequency selectivity,” *J. Acoust. Soc. Am*. 112, 1145-1157.

TABLE S1
ACOUSTIC ANALYSES

Condition	Unenhanced	Enhanced	Reduction
Quiet- Moderate	3.49 (0.025)	1.73 (0.011)	50.5%
Quiet- Severe	4.91 (0.031)	2.51 (0.012)	48.9%
Noise- Moderate	3.84 (0.016)	1.86 (0.012)	51.7%
Noise- Severe	5.32 (0.024)	2.74 (0.018)	48.5%

The amount by which the excitation pattern for a sample vowel (/ε/) had to be broadened (*re*: normal) in order to match individual smeared frames under different forms of processing. The numbers in the parentheses represent the standard error across 10 repetitions (phase randomizations). The last column is the percent by which enhancement reduced the bandwidth of the best-fitting excitation pattern relative to its unenhanced complement.

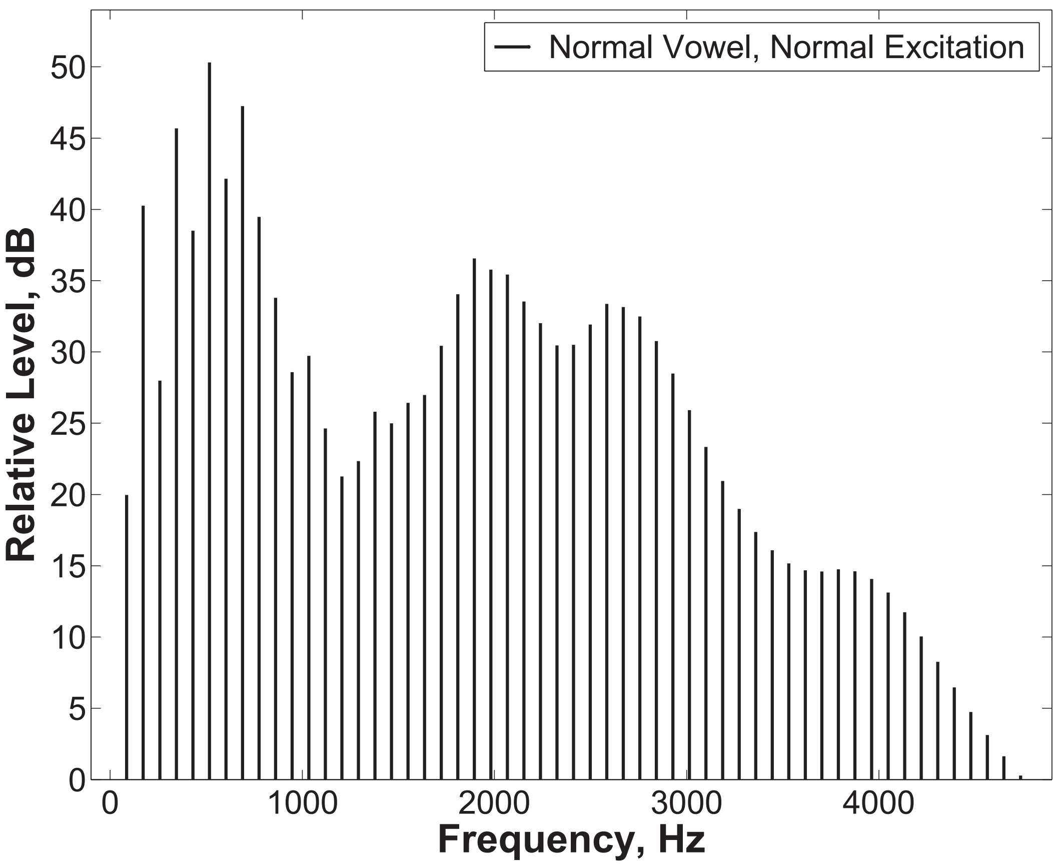

Fig. S2. Excitation pattern of the normal, un-smeared vowel.

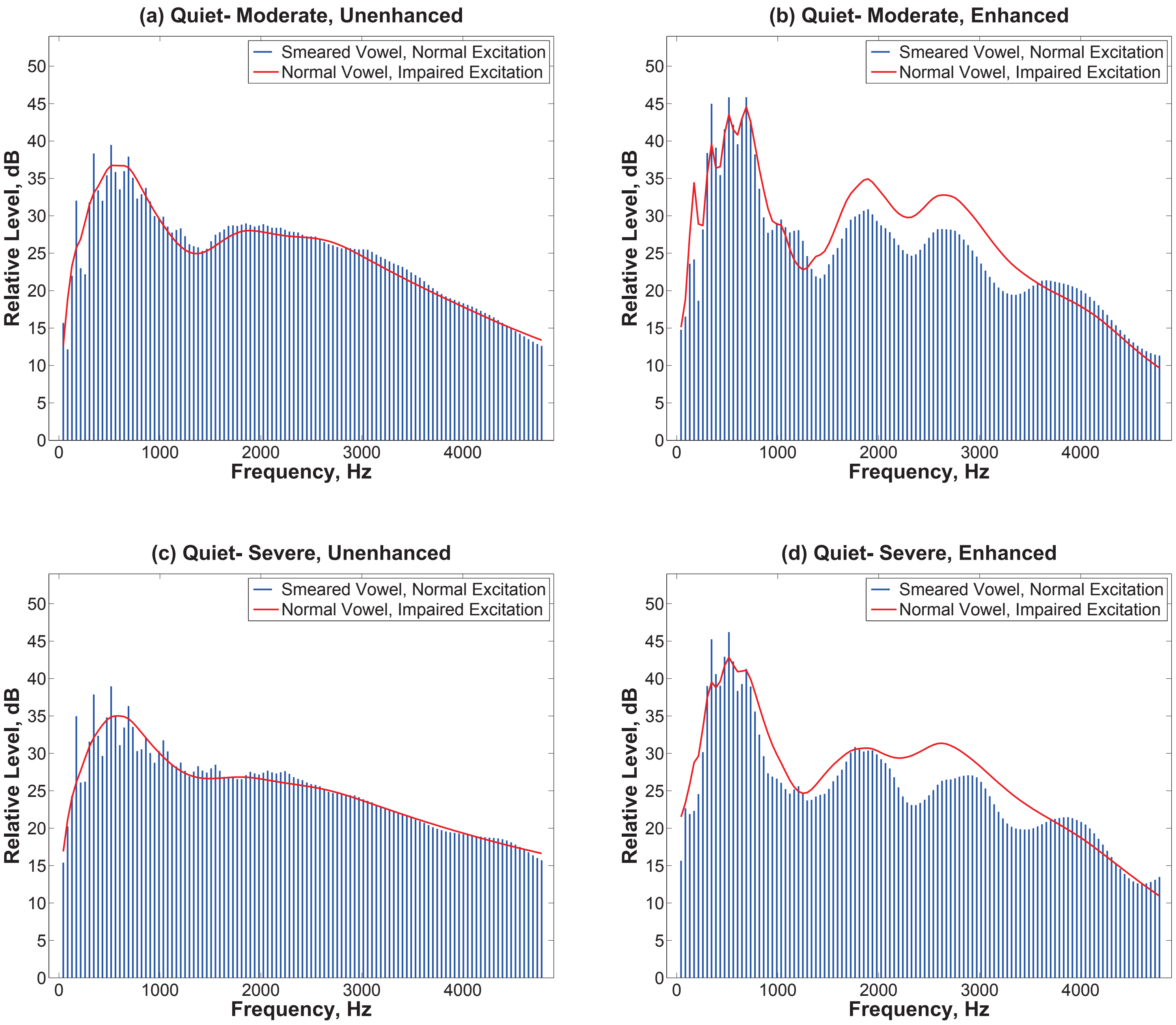

Fig. S3. Acoustic analyses for the vowel in quiet. Blue bars show the excitation patterns of the smeared vowels and the red lines show the least-squares fit of the impaired excitation pattern for the normal vowel. Panels (a) and (c) show excitation patterns for the unenhanced vowels and panels (b) and (d) show the excitation patterns for the enhanced vowels. Panels (a) and (b) of each figure represent a moderate amount of spectral smearing and panels (c) and (d) represent a severe amount of smearing.

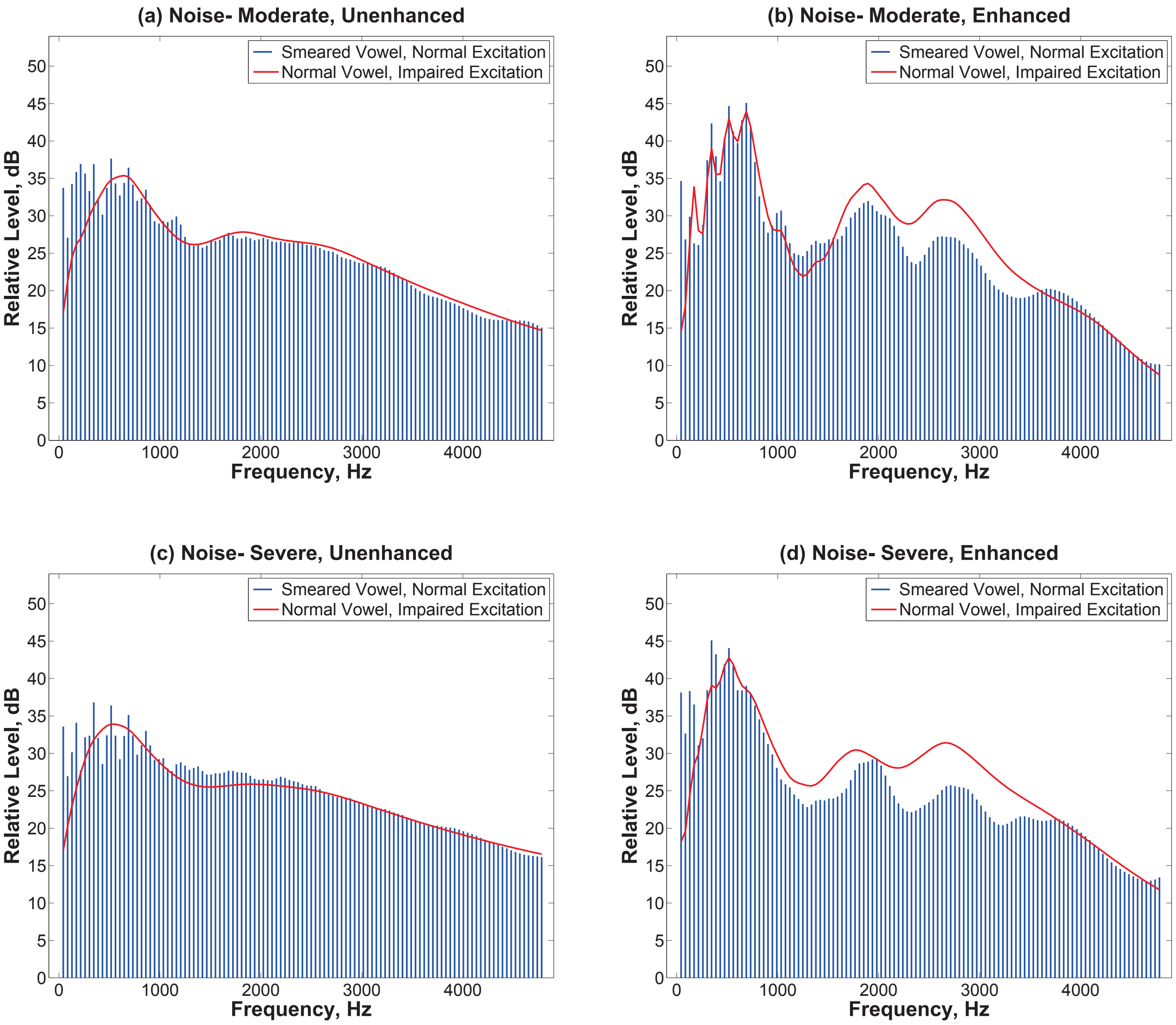

Fig. S4. Acoustic analyses for the vowel in noise. See Figure S3 legend.
